# Integrating Transcriptomics and Gut Microbiota Analysis Reveals Adaptive Mechanisms of Alkaline Stress on the Molting and Intestinal Immune Responses in Pacific White Shrimp, *Litopenaeus vannamei*

**DOI:** 10.3390/life16040652

**Published:** 2026-04-12

**Authors:** Yiming Li, Yucong Ye, Junling Ma, Zongli Yao, Yan Li, Pengcheng Gao, Yuxin Wang, Zihe Cheng, Yunlong Zhao, Qifang Lai

**Affiliations:** 1 East China Sea Fisheries Research Institute, Chinese Academy of Fishery Sciences, Shanghai 200090, China; 2 School of Life Science, East China Normal University, Shanghai 200241, China; 3 College of Fisheries and Life Science, Shanghai Ocean University, Shanghai 201306, China

**Keywords:** alkaline stress, *Litopenaeus vannamei*, oxidative damage, transcriptional regulation, intestinal immunity

## Abstract

In northwestern China, there is an abundance of saline-alkali water resources, but their high alkalinity severely restricts the development of inland saline-alkali water aquaculture. As an important aquaculture species, the whiteleg shrimp, *Litopenaeus vannamei*, shows an unclear physiological adaptation mechanism under high-alkaline stress. In this study, multi-omics and physiological methods were used to systematically reveal the effects of high-alkaline stress on the molt, antioxidation response, and immune defense in *L. vannamei*. The results showed that high-alkaline stress caused damage to the intestinal tissues of the shrimp and weakened the mucous barrier function, which was accompanied by a significant decrease in the activities of antioxidant enzymes (SOD, CAT, and GPx) and non-specific immune indicators (PO and LZM) (*p* < 0.05). The transcriptome results showed that the expression of genes related to chitin metabolism and calcium ion binding was upregulated, whereas that of genes related to muscle contraction and cell skeleton construction was downregulated. The structure of the intestinal microbiota changed significantly, with a decrease in microbiota diversity, whereas the abundance of potential pathogenic species (e.g., *Photobacterium*) increased. These results provide a theoretical basis for clarifying the molting response and antioxidant defense mechanism of *L. vannamei* in high-alkaline environments, with significance for saline-alkali water aquaculture practices.

## 1. Introduction

China has 991.3 million ha of saline-alkali land, of which ~55% (45.87 million ha) is saline-alkali surface water [1]. Saline-alkali fisheries have a significant impact on the ecological restoration and management of abandoned saline-alkali water areas and are gaining in popularity [2]. However, to support their successful economic development, it is vital to study the physiological responses of aquatic organisms to such alkaline environments.

Saline-alkali water is characterized by a high pH value, a high ionic coefficient, and an imbalance of major ions, resulting in extremely low utilization rates [3]. The high-alkaline pressure in saline-alkali water can have complex physiological effects on aquatic organisms, such as disrupting their internal ion balance and causing oxidative damage [4]. It was also shown to cause significant damage to the gills and hepatopancreas of *L. vannamei*, accompanied by cell apoptosis [2,5]. High alkalinity (pH > 9) affects the hemolymph of the olive flatfish *Palichthys olivaceus*, inducing changes in the activities of antioxidant enzymes (SOD and GST), and significantly increasing the level of immunoglobulin M [6]. Long-term exposure to alkaline conditions interferes with glycolysis in *Carassius auratus*, triggering immune–inflammatory responses and cell apoptosis [7].

Molting is an important biological process in the growth and development of shrimp [8]. Successful molting determines the resulting exoskeleton of crustaceans, which is crucial for their growth and defense [9]. This requires the coordinated expression of multiple genes, among which those encoding molting hormones, such as EH and ETH, have a crucial role [10]. The characteristics of crustacean exoskeletons are influenced by various environmental factors, such as pH value, pollutants and ion concentrations, among others [11]. A high pH environment systematically disrupts absorption and distribution in *L. vannamei*. This disruption is primarily driven by membrane transporter inhibition and oxidative tissue damage, which ultimately interfere with molting and exoskeleton reconstruction [11]. Furthermore, extreme alkaline stress dramatically shifts the ambient acid-base balance and osmotic pressure. Aquatic organisms exposed to these environmental fluctuations experience compromised ionic homeostasis, subsequently triggering severe oxidative stress [12]. For instance, high carbonate alkalinity inhibits the antioxidant immune system of carp gill tissues and induces cell apoptosis [13]. In *L. vannamei* under high-alkaline stress, the antioxidant defense system is activated to cope with the oxidative stress induced by the high-alkaline environment [14]. This is supported by the regulation of energy metabolism to enable the shrimp to maintain ion and acid–base homeostasis and alleviate oxidative stress under alkaline conditions [15].

Most marine aquatic crustaceans maintain the balance of their body fluids with their external environment and adapt through osmosis [16]. Maintaining ion homeostasis is also vital for fluid balance and energy metabolism [17]. Gills are not only important respiratory organs, but also function in ion balance and osmoregulation [18]. Na^+^/K^+^-ATPase in the gills is crucial for osmotic regulation, having a significant role in both high and low osmotic regulation. V(H^+^)-ATPase also has an important role in osmotic regulation [19]. Acute alkaline stress can cause ion imbalances in aquatic organisms and even lead to alkalosis [7]. The alkaline pH value causes an increase in mucus secretion, which affects osmoregulation and results in an increase in the concentrations of Na^+^ and HCO^3−^ in the blood, increasing osmotic pressure [20]. It was found that the activity of ion transport-related enzymes in *L. vannamei* decreased under high-alkaline stress, which might be caused by the accumulation of ROS induced by such stress. This stress attacked the sulfhydryl groups of the ion transport enzymes, disrupting their conformation and catalytic activity [14]. Thus, under acid–base stress, the gills and intestines of *L. vannamei* trigger the transport of different ions to promote the osmotic balance [5,18].

The intestines of crustaceans are important organs for permeation regulation and have a significant role in the immune process [21]. Under high-alkaline stress, crustaceans regulate their physiology by maintaining intestinal homeostasis [22]. The gut microbiota is vital in regulating homeostatic mechanisms by helping to establish the intestinal epithelial barrier and maintaining the immune homeostasis of the host [23]. Extensive secondary metabolites produced by the symbiotic gut microbiota are crucial for the regulation of the physiology and immune response of their hosts [24]. However, the gut microbiota is subject to environmental selection, affected by factors such as the immune system, intestinal pH, and diet of their host [25,26]. High-alkaline stress causes damage to the intestine of *L. vannamei*, decreasing the expression of immune markers and reducing symbiotic bacteria related to polysaccharide metabolism, energy utilization, and intestinal immunity [14]. High-alkaline stress can also alter the structure and function of the intestinal microbial community by increasing the abundance of pathogenic bacteria in the intestine [27], indirectly interfering with the metabolic regulatory network of aquatic organisms [28].

*L. vannamei* is an important species in the aquaculture industry worldwide, with delicious meat, high nutritional value and rapid growth rates [29]. Although it is currently farmed on a small scale in inland saline-alkali waters in northwest China, it is a promising economic species for the development of saline-alkali fisheries [30,31]. However, its alkaline tolerance is unstable, with its survival rate decreasing significantly when the pH exceeds 8.3 [32]. While previous studies have provided some insights into how this species responds to high alkalinity, further information is required to enable the successful aquaculture of this species in saline-alkali waters. Therefore, this study investigated the molting process and antioxidant defense mechanisms of *L. vannamei* under high-alkaline conditions, providing a theoretical basis for the development of shrimp agriculture in such environments.

## 2. Materials and Methods

### 2.1. Shrimp Husbandry, Acclimation, and Carbonate Alkalinity Exposure

The *L. vannamei* used in this study were collected from an aquaculture facility in Fengxian District, Shanghai, transported to the Aquatic Organism Breeding Station of the East China Sea Fisheries Research Institute, Chinese Academy of Fishery Sciences, and acclimated for 1 week in multiple 300 L aquaria. During rearing, several PVC pipes were placed in each aquarium as shelters to reduce cannibalism during molting. Nets were provided as attachment substrates for adult shrimp. Water quality was maintained at 20 ± 1 °C, pH 7.8 ± 0.1, salinity 5‰, dissolved oxygen (DO) 6.23 ± 0.04 mg/L, and ammonia nitrogen ≤ 0.1 mg/L. Shrimp were fed to apparent satiation four times daily (08:00 h, 12:00 h, 17:00 h, and 22:00 h) with a commercial diet (Guangdong Haid Group Co., Ltd., Guangzhou, Guangdong, China), with a total daily ration equivalent to 5% of the shrimp biomass. Throughout the experiment, water was renewed daily, and residual feed, dead shrimp, and feces were promptly removed.

Based on our previous study on acute carbonate alkalinity stress in *L. vannamei*, five carbonate alkalinity levels were established: 2.5 (control, Lv-C), 5, 10, 20, and 40 mmol/L. This gradient was deliberately designed to encompass both environmentally realistic alkaline fluctuations (5–10 mmol/L) and extreme acute stress conditions (20–40 mmol/L). Testing these higher concentrations is crucial for delineating the physiological tolerance limits of the shrimp of inland saline-alkali waters. Each treatment included 30 healthy adult shrimp, which were exposed to the designated alkalinity for 96 h. During exposure, alkalinity was measured every 12 h by acid–base titration with 0.1 mol/L hydrochloric acid (HCl) and 0.1 mol/L sodium hydroxide (NaOH) used to maintain the pH and alkalinity of the water. At the end of the 96 h exposure, survival was recorded for each group, and final body length and body weight were measured (Table 1). After anesthesia by immersion in ice water, five shrimp were randomly selected from both the control group (Lv-C) and the 20 mmol/L group (treatment group, Lv-T), and hemolymph was collected using disposable syringes to evaluate innate immune parameters. For transcriptomic analysis, gills were collected from nine shrimp per group (control and treatment), and tissues from three shrimp were pooled to form one biological replicate (*n* = 3 replicates per group). For gut microbiota analysis, intestines were collected from ten shrimp per group, and tissues from two shrimp were pooled to constitute one biological replicate (*n* = 5 replicates per group). All sampled tissues were immediately snap-frozen in liquid nitrogen. In addition, intestinal tissues from an additional three shrimp from both the control and treatment groups were aseptically excised using sterile scissors for morphological analysis. The results of growth indicators were shown in Table 1.

### 2.2. Histological and Histochemical Analysis

#### 2.2.1. Sample Preparation and H&E Staining

Midgut segments of *L. vannamei* from the control and treatment groups were dissected on ice and gently rinsed with sterile PBS (pH 7.4) to remove external hemolymph and debris without disturbing the luminal contents. The tissues were fixed in 4% paraformaldehyde (Servicebio, Wuhan, China) at 4 °C for 24 h. Following fixation, the specimens were dehydrated through a graded ethanol series, cleared in xylene, and embedded in paraffin. Serial sections were cut at a 5 µm thickness and mounted on glass slides. The sections were deparaffinized, rehydrated, and stained with hematoxylin and eosin (H&E) following standard histological protocols to evaluate general tissue morphology.

#### 2.2.2. Alcian Blue Staining

To assess mucus secretion, 5 µm paraffin sections were deparaffinized in xylene and rehydrated through a graded ethanol series to distilled water. The sections were incubated with Alcian Blue (AB) solution (pH 2.5) for 30 min at room temperature to visualize acidic mucopolysaccharides (AMPs). After rinsing with distilled water, the sections were counterstained with Mayer’s hematoxylin, washed in running tap water for 5 min, dehydrated, cleared in xylene, and mounted with a neutral resin mounting medium.

#### 2.2.3. Wheat Germ Agglutinin Immunofluorescence

For the visualization of the peritrophic membrane (PM), midgut segments (5 mm) were fixed in 4% paraformaldehyde at 4 °C for 2–4 h and washed with PBS. The samples were cryoprotected in 30% sucrose overnight, embedded in OCT compound, and frozen in liquid nitrogen. Cryosections (5 µm) were prepared and mounted on adhesive slides. The sections were washed with PBS and incubated with fluorescein-labeled succinylated wheat germ agglutinin (sWGA-FITC, 25 µg/mL) for 30 min in the dark. Nuclei were counterstained with Hoechst 33342 (10 µg/mL) (Beyotime Biotechnology, Shanghai, China) for 15 min. Finally, the sections were washed, mounted with an antifade mounting medium, and imaged using a fluorescence microscope (Leica DMi8, Leica Microsystems GmbH, Wetzlar, Germany).

### 2.3. Basic Immunological Parameters

The phagocytic rate (PR) was determined by placing 100 μL of diluted hemolymph from each sample (five shrimp per treatment group; *n* = 5) onto a glass slide and incubating at room temperature for 1 h to allow for hemocyte adhesion. The slides were gently rinsed with Hank’s balanced salt solution (HBSS) to remove non-adherent cells. Adherent hemocytes were stained with 5% Giemsa for 20 min and then fixed with 200 μL methanol before microscopic examination. The PR was defined as the proportion of phagocytic (particle-ingesting) hemocytes among the total hemocytes counted and was calculated using Equation (1):

PR (%) = (number of phagocytic hemocytes/total hemocytes observed) × 100.(1)

Each treatment included five biological replicates, and each biological replicate was assessed in triplicate as technical replicates.

The respiratory burst (RB) activity of hemocytes was measured using the nitroblue tetrazolium (NBT) reduction assay following established protocols for penaeid shrimp and crustacean hemocytes [33,34]. Briefly, 100 μL of hemolymph from each shrimp was placed in a 96-well microplate and allowed to adhere for 30 min. The hemocytes were then incubated with 100 μL of 0.2% NBT solution for 30 min at room temperature to allow for phagocytosis and intracellular reduction. After washing twice with HBSS to remove unreacted NBT, the cells were fixed with absolute methanol. The intracellular formazan crystals were subsequently solubilized by adding 120 μL of 2 M KOH and 140 μL of DMSO per well. The optical density of the triplicate wells was recorded at 630 nm using a microplate reader (Model 680; Bio-Rad Laboratories, Hercules, CA, USA).

### 2.4. Enzyme Activity Assays

Intestinal tissues were collected from five shrimp per treatment group (*n* = 5), with five biological replicates and three technical replicates for each treatment. The tissues were transferred into sterile centrifuge tubes, thoroughly homogenized by mixing and shaking, and then diluted with 0.86% physiological saline at a tissue:saline ratio of 1:9 (*w*/*v*). The homogenates were centrifuged at 4 °C and 2500 rpm for 20 min, after which the supernatants were carefully collected for subsequent analyses. Commercial assay kits for superoxide dismutase (SOD), catalase (CAT), glutathione peroxidase (GPX), reduced glutathione (GSH), phenoloxidase (PO), and lysozyme (LZM) (Jiangsu Nanjing Jiancheng Biotechnology Co., Ltd., Nanjing, China) were used to quantify intestinal enzyme activities in accordance with the manufacturer’s instructions.

### 2.5. Gene Expression Analysis

Total RNA was extracted from each group using TRIzol reagent (Aidlab, Beijing, China) according to the manufacturer’s instructions. First-strand cDNA was synthesized by reverse transcription and subsequently used as the template for quantitative real-time PCR (qRT-PCR). qRT-PCR was performed using Vazyme (Nanjing, China) SYBR Green in a 20 μL reaction system with gene-specific primers. The thermal cycling program was as follows: initial denaturation at 95 °C for 1.5 min, followed by 40 cycles of denaturation at 95 °C for 10 s and annealing/extension at 60 °C for 30 s. A melting-curve analysis was conducted from 65 °C to 95 °C to confirm amplification specificity. Three shrimp were analyzed per treatment group (*n* = 3), with three biological replicates and three technical replicates for each treatment. β-actin (AF300705.2) was used as the internal reference gene for normalization, and relative expression levels were calculated using the 2^−ΔΔCt^ method [35]. The primer sequences are provided in Table 2.

### 2.6. Transcriptome Sequencing and Analysis

After 96 h of exposure, gill tissues were sampled from the control (Lv-C) and high-alkalinity (Lv-T) groups. Nine shrimp per group were collected, and tissues from three individuals were pooled to form one biological replicate (*n* = 3). Samples were snap-frozen in liquid nitrogen and stored at −80 °C until use. Total RNA was extracted using TRIzol reagent (Invitrogen™, Thermo Fisher Scientific Inc., Waltham, MA, USA), and quality was assessed using a NanoDrop™ 2000 spectrophotometer (Thermo Fisher Scientific Inc., Waltham, MA, USA) and Agilent 2100 Bioanalyzer (Agilent Technologies, Inc., Santa Clara, CA, USA). Libraries were constructed using the VAHTS Universal V6 RNA-seq Library Prep Kit (Vazyme Biotech Co., Ltd., Nanjing, Jiangsu, China) and sequenced on an Illumina NovaSeq 6000 platform (Illumina, Inc., San Diego, CA, USA).

Raw data were filtered to remove low-quality reads and adapters using fastp (version 0.20.0). Clean reads were aligned to the *L. vannamei* reference genome using HISAT2. Gene expression levels were quantified as FPKM using HTSeq-count. Principal component analysis (PCA) and differential expression analysis (Lv-T vs. Lv-C) were performed in R using the DESeq2 package. Genes with an adjusted *p*-value (q-value) < 0.05 and |log2FoldChange| > 1 were identified as differentially expressed genes (DEGs). Gene Ontology (GO) and Kyoto Encyclopedia of Genes and Genomes (KEGG) enrichment analyses were performed using a hypergeometric test. Gene Set Enrichment Analysis (GSEA) was conducted on a pre-ranked gene list based on Equation (2):

−log10(*p*-value) × sign(log2FoldChange). (2)

### 2.7. Intestinal Microbiota Sequencing and Analysis

Intestinal contents were collected from the Lv-C and Lv-T groups (*n* = 5; each replicate pooled from ten individuals). Genomic DNA was extracted using the MagPure Tissue DNA LQ Kit (Magen Biotechnology, Guangzhou, China). The V3-V4 region of the bacterial 16S rRNA gene was amplified using primers 341F and 806R with Takara Ex Taq polymerase (Takara Bio Inc., Kusatsu, Japan). PCR was performed using the following thermal cycling program: 95 °C for 3 min; 25 cycles of 95 °C for 30 s, 55 °C for 30 s, and 72 °C for 30 s; followed by a final extension at 72 °C for 5 min. PCR products were purified with AMPure XP beads (Beckman Coulter, Brea, CA, USA), indexed, and sequenced on an Illumina NovaSeq 6000 platform (Illumina, Inc., San Diego, CA, USA).

Sequence data were processed using the QIIME2 pipeline (v2020.11). Denoising, paired-end merging, and chimera removal were performed using DADA2 to infer amplicon sequence variants (ASVs). Taxonomic assignment was performed using the q2-feature-classifier against the SILVA database (v138). Alpha diversity indices (e.g., Shannon and Chao1) and beta diversity metrics (e.g., Bray–Curtis and weighted UniFrac) were calculated in QIIME2. Differences in community structure were evaluated using PERMANOVA and ANOSIM in R (version 4.3.2). Differentially abundant taxa were identified using LEfSe (version 1.1.2) with an LDA score threshold > 2.0.

### 2.8. Statistical Analysis

Quantitative data are expressed as mean ± standard deviation (SD). Statistical analyses were conducted using SPSS Statistics v22.0 (IBM, Armonk, NY, USA), implementing a hierarchical analytical framework: For comparisons across different alkalinity treatments, one-way ANOVA with Duncan’s multiple range test was applied for post hoc intergroup comparisons (α = 0.05). Intergroup differences between the Lv-C and Lv-T groups were assessed through independent two-tailed Student’s *t*-tests, with significance at *p* < 0.05.

## 3. Results

### 3.1. High-Alkalinity Stress Suppresses Antioxidant Defenses and Hemocyte Immune Functions in L. vannamei

**Figure 1:**
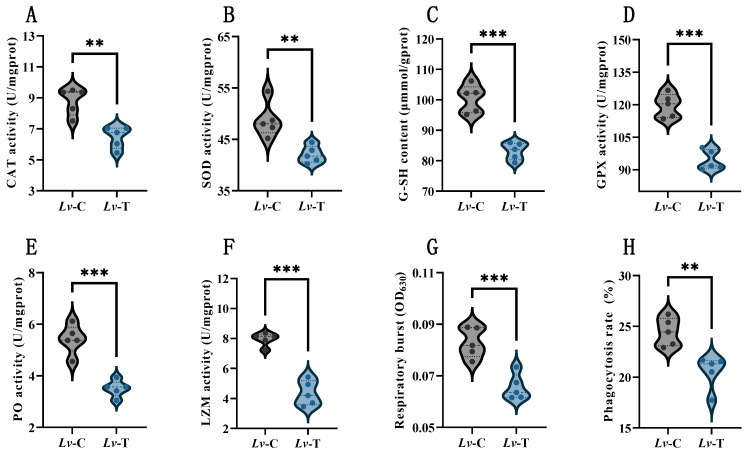
Effects of high-alkalinity stress on antioxidant and innate immune parameters in *L. vannamei*. (**A**) CAT; (**B**) SOD; (**C**) GSH; (**D**) GPX; (**E**) PO; (**F**) LZM; (**G**) RB; and (**H**) PR. Data are expressed as mean ± SD (*n* = 5). Asterisks indicate significant differences between the treatment (Lv-T) and control (Lv-C) groups (** *p* < 0.01; *** *p* < 0.001). Violin plots illustrate the distribution and probability density of the individual data points (black and blue dots) for each group. Horizontal dashed lines within the violins represent the median and the upper/lower quartiles.

As shown in Figure 1, in terms of antioxidant and detoxification capacity, SOD and CAT activities in the Lv-T group were significantly lower than those in the Lv-C group (*p* < 0.01). GPx activity and reduced GSH content were also markedly decreased (*p* < 0.001), suggesting that the glutathione-dependent peroxide-reduction pathway could not be maintained at a high operating level under prolonged stress.

In terms of immune-related enzyme activities, PO activity in Lv-T was lower than in Lv-C (*p* < 0.001), consistent with suppression of the proPO–PO cascade and/or reduced effective activation resulting from compromised hemocyte status under chronic stress. LZM activity was also significantly reduced (*p* < 0.001), implying a weakened humoral capacity to lyse bacterial cell-wall components. In terms of hemocyte function, both the RB and the PR were significantly decreased in Lv-T compared with Lv-C (*p* < 0.01), indicating that phagocytic uptake and lysosomal processing were jointly constrained by energy reallocation and disruption of cellular homeostasis.

### 3.2. Alkalinity Stress Differentially Modulates Immune-Related Gene Expression in L. vannamei

**Figure 2:**
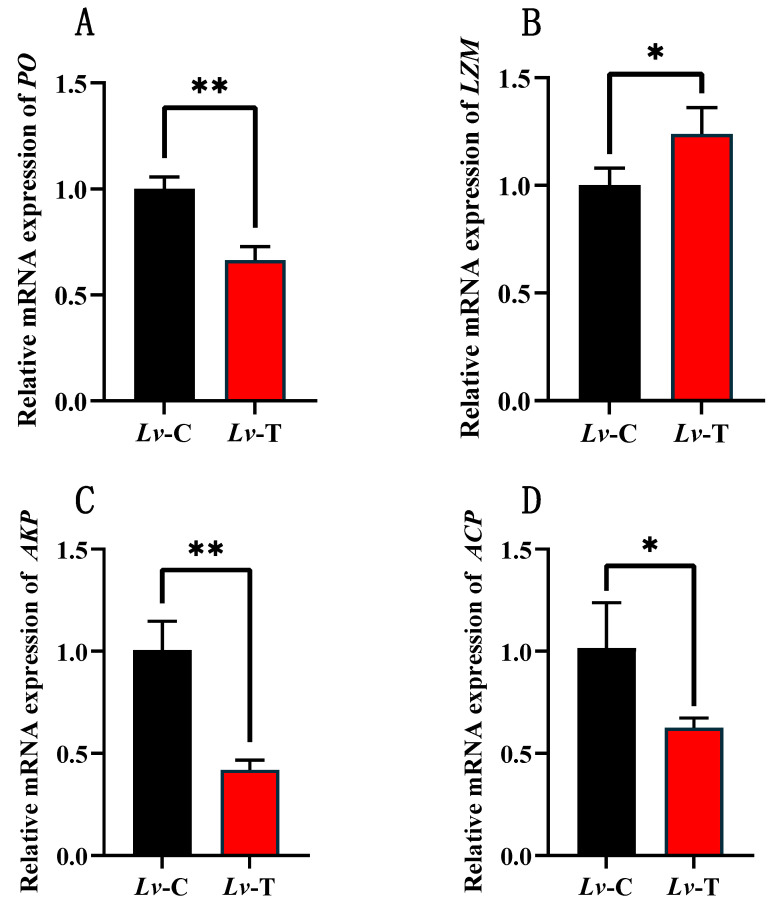
Changes in the expression of genes encoding immune enzymes in *L. vannamei* under high-alkalinity stress: (**A**) *phenoloxidase* (*PO*); (**B**) *lysozyme* (*LZM*); (**C**) *alkaline phosphatase* (*AKP*); and (**D**) *acid phosphatase* (*ACP*). Data are means ± SE (*n* = 3); * *p* < 0.05; ** *p* < 0.01.

As shown in Figure 2, following alkalinity stress, the expression of immune-related genes also changed markedly. Compared with the Lv-C group, transcripts of *PO*, *AKP*, and *ACP* were significantly downregulated in the Lv-T group (*p* < 0.05). By contrast, *LZM* expression was significantly upregulated in Lv-T (*p* < 0.05), suggesting that enhancing antibacterial defense against external pathogen invasion would be particularly important under alkalinity stress.

### 3.3. Histological and Histochemical Analyses

**Figure 3:**
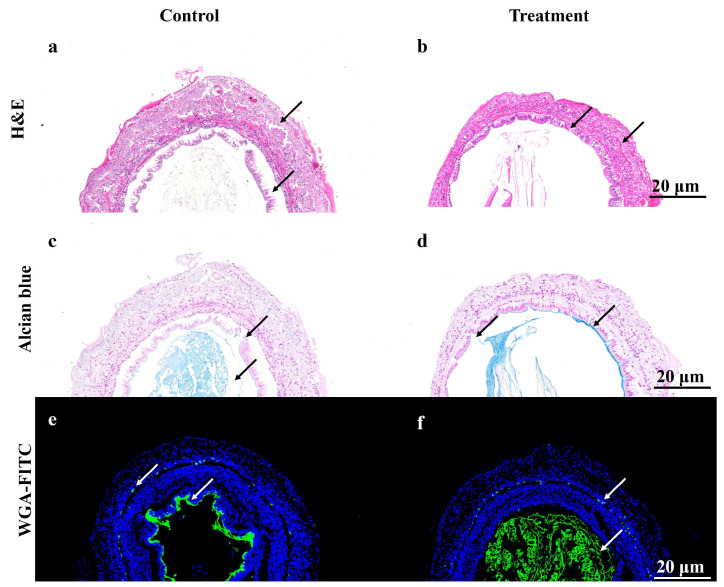
Histological analysis of the intestinal tissues of *L. vannamei* under high-alkalinity stress. (**a**,**b**) Hematoxylin and eosin (H&E) staining, where black arrows indicate the intact, continuous brush border in the control group (**a**) and the shrunken, disorganized epithelial layer in the treatment group (**b**). (**c**,**d**) Alcian Blue (AB) staining, where black arrows highlight abundant acidic mucopolysaccharide secretion (blue) in the control (**c**) and severely depleted mucin in the treatment group (**d**). (**e**,**f**) Wheat germ agglutinin (WGA) fluorescence staining (green), where white arrows indicate the continuous peritrophic membrane in the control (**e**) and its fragmented dispersal in the treatment group (**f**).

As shown in Figure 3, the histological results indicated that, compared with the control group, shrimp reared under high carbonate alkalinity conditions exhibited marked histological alterations in the midgut. In the control group, the midgut displayed a normal and well-organized architecture. The basal lamina remained intact, and the epithelial layer was tightly connected to the underlying muscularis, with a dense and continuous brush border (microvilli) facing the lumen. By contrast, exposure to high alkalinity induced severe histopathological damage to the midgut tissue. The brush border lost its uniform appearance and showed pronounced shrinkage with a rugose (wrinkled) morphology. Compared with the control, the microvilli appeared shorter and more irregular, and the muscle fibers exhibited signs of constriction, accompanied by reduced interstitial spaces relative to the relaxed muscle layer observed in the control group.

To evaluate the impact of high alkalinity on the mucosal barrier, AB staining was performed to detect AMPs. In the control group, abundant AB-positive signals (blue staining) were distributed along the apical surface of the epithelium and within the secretory vesicles of epithelial cells, indicating active mucus secretion and a robust chemical barrier. However, in the high alkalinity group, both the intensity and coverage of AB-positive staining were markedly reduced. The continuous mucus layer covering the apical surface was disrupted and appeared fragmented, suggesting that sustained alkalinity stress compromised mucus secretion and/or inhibited the synthesis of acidic mucins in the midgut epithelium.

WGA labeling was used to visualize *N*-acetylglucosamine residues, primarily marking the PM and the glycocalyx. In the control group, WGA fluorescence showed a strong, continuous, and linear signal lining the luminal side, consistent with a well-formed PM, together with distinct staining along the brush border. By contrast, the high alkalinity group exhibited severe disruption of the PM. The characteristic linear definition of the PM was lost, and the midgut lumen contained diffuse and disorganized WGA-positive signals (green fluorescence). This widespread fluorescence suggests extensive fragmentation of the PM, with dispersal of chitinous components and associated glycoproteins throughout the luminal space, indicating profound impairment of the physical barrier function under alkalinity stress.

### 3.4. Alkaline Stress Induced Transcriptomic Changes in L. vannamei

**Figure 4:**
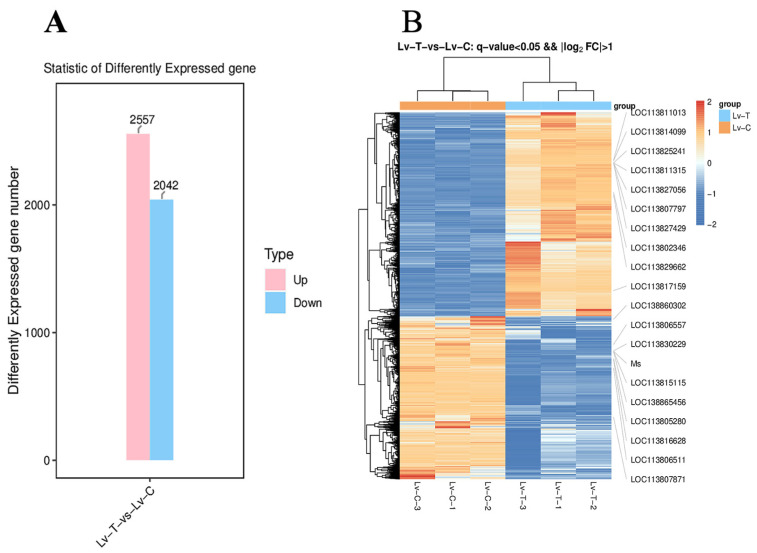
Differentially expressed genes (DEGs) identified in *L. vannamei*. (**A**) DEGs between the treatment and control groups (Lv-T and Lv-C, respectively). Pink columns represent significantly upregulated genes, and blue columns represent significantly downregulated genes. (**B**) Cluster diagram of DEGs. Red indicates genes encoding proteins with relatively high expression levels, whereas blue represents genes encoding proteins with relatively low expression levels.

**Figure 5:**
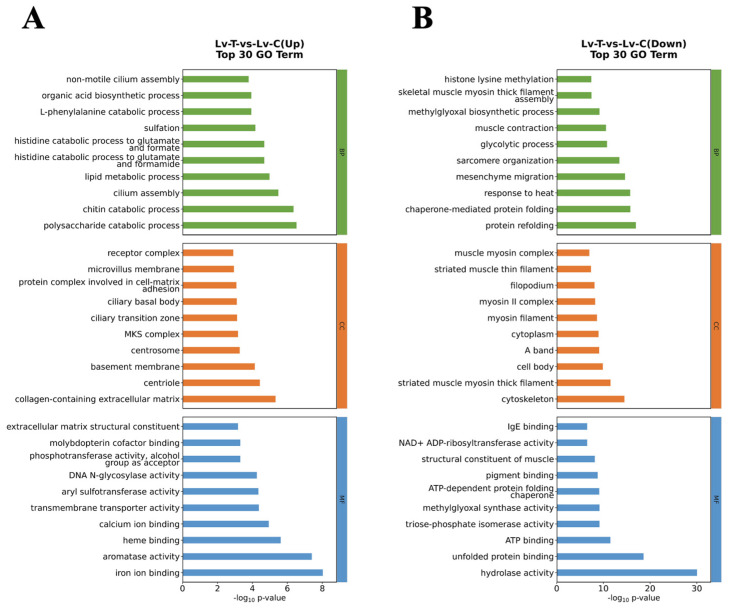
Gene function classification in GO analysis of *L. vannamei*. (**A**) The top 20 enriched gene functions of the upregulated genes; (**B**) the top 20 enriched gene functions of the downregulated genes. BP, biological process; CC, cellular component; MF, molecular function.

After 96 h of exposure to alkaline conditions, *L. vannamei* exhibited extensive transcriptional reprogramming relative to the control group. In total, 4599 DEGs were detected, comprising 2557 upregulated and 2042 downregulated genes (Figure 4A). Hierarchical clustering showed a robust treatment-driven separation, with replicates from the same group clustering closely, indicating high within-group consistency (Figure 4B). GO enrichment analysis revealed that upregulated DEGs were primarily associated with calcium ion binding (GO:0005509), chitin catabolic processes (GO:0006032), and transmembrane transporter activity (GO:0022857) (Figure 5A). By contrast, downregulated DEGs were mainly enriched in functions linked to muscle contraction (GO:0006936), cytoskeleton (GO:0005856), protein refolding (GO:0042026), and ATP binding (GO:0005524) (Figure 5B).

**Figure 6:**
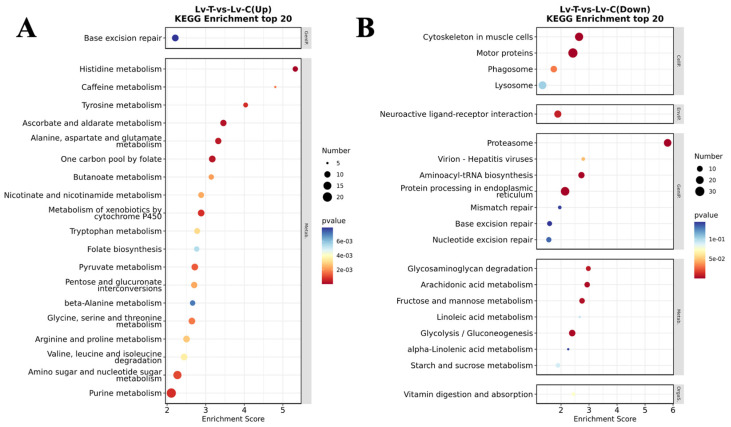
KEGG pathway enrichment classification of *L. vannamei*. (**A**) The top 20 enriched pathways of the upregulated genes; (**B**) the top 20 enriched pathways of the downregulated genes.

Consistent with the GO patterns, KEGG analysis indicated that upregulated genes were significantly overrepresented in amino sugar and nucleotide sugar metabolism (pvm00520), which is central to chitin production and cuticle formation during molting (Figure 6A). Meanwhile, downregulated genes were enriched in neuroactive ligand–receptor interaction (pvm04080), a pathway involving chloride ion transport, and cytoskeleton in muscle cells (pvm04820), suggesting muscle structural remodeling (Figure 6B).

**Figure 7:**
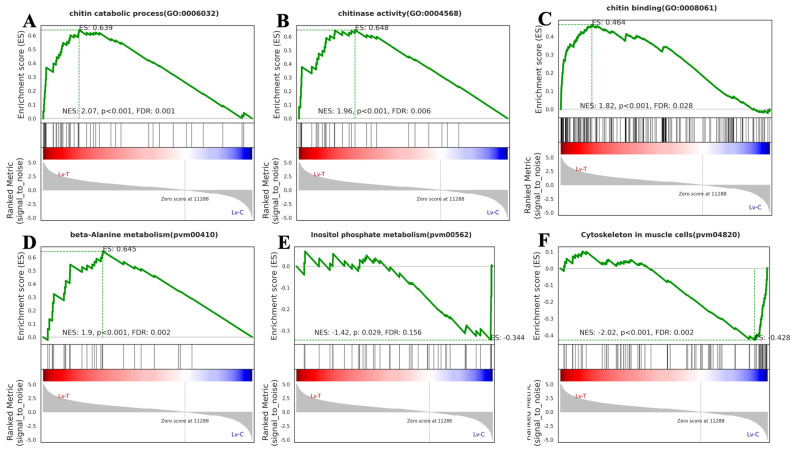
GSEA of *L. vannamei* in the high alkalinity group. (**A**) Chitin catabolic process (GO:0006032); (**B**) chitinase activity (GO:0004568); (**C**) chitin binding (GO:0008061); (**D**) beta-alanine metabolism (pvm00410); (**E**) inositol phosphate metabolism (pvm00562); and (**F**) cytoskeleton in muscle cells (pvm04820). NES, Nominal *p*-value, and FDR were determined using GSEA software and are indicated within each enrichment plot.

To further resolve pathway-level changes, GSEA based on GO and KEGG annotations was conducted. Gene sets related to chitin catabolic process (GO:0006032), chitinase activity (GO:0004568), chitin binding (GO:0008061), and beta-alanine metabolism (pvm00410) were significantly upregulated (Figure 7A–D), whereas inositol phosphate metabolism (pvm00562) and cytoskeleton in muscle cells (pvm04820) were significantly downregulated (Figure 7E, F).

### 3.5. Intestinal Microbial Diversity in L. vannamei

**Figure 8:**
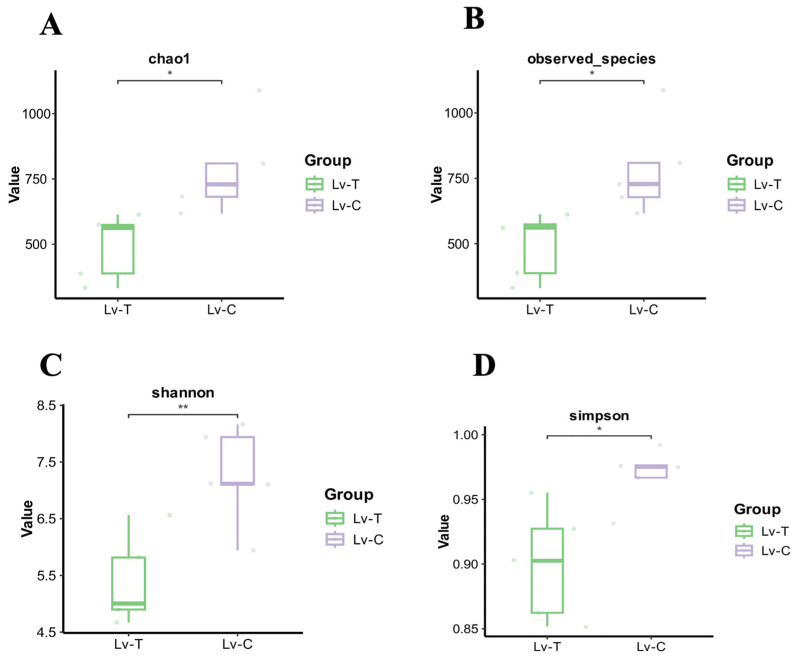
Alpha diversity indices of the intestinal microbiota of *L. vannamei*. (**A**) Chao1 richness index; (**B**) observed species; (**C**) Shannon diversity index; and (**D**) Simpson diversity index. * *p* < 0.05; ** *p* < 0.01.

As shown in Figure 8, to evaluate the effects of an alkaline environment on the richness and diversity of the intestinal microbiota in *L. vannamei*, multiple alpha diversity indices were calculated. Compared with the control Lv-C group, the Chao1 index of the damaged Lv-T group was significantly decreased (*p* < 0.05). Consistently, the number of observed taxa in the Lv-T group was also significantly lower than that in the Lv-C group (*p* < 0.05). Similarly, the Shannon and Simpson indices in the Lv-T group were significantly lower than those in the control group, indicating a pronounced reduction in both species richness and evenness (*p* < 0.05).

**Figure 9:**
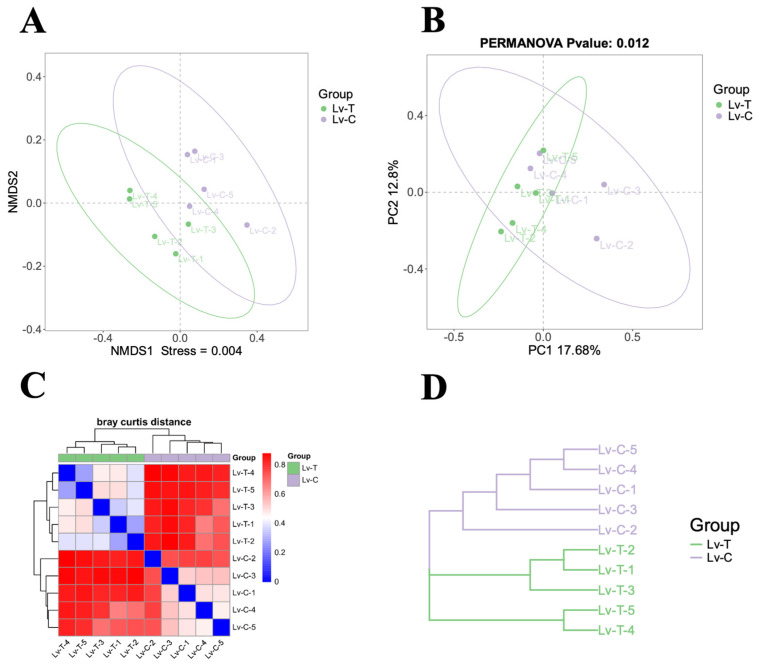
Beta diversity of the intestinal microbiota in *L. vannamei* under high-alkalinity stress. (**A**) NMDS and (**B**) PCoA ordination plots. (**C**) Bray–Curtis distance matrix heatmap. (**D**) UPGMA hierarchical clustering tree.

As shown in Figure 9, non-metric multidimensional scaling (NMDS) and principal coordinate analysis (PCoA) ordination plots showed that samples from the Lv-T group were clearly separated from those of the control Lv-C group in 2D space. PERMANOVA (Adonis) yielded R^2^ = 0.4989 and *p* = 0.012, and ANOSIM analysis produced R = 0.924 and *p* = 0.013, demonstrating that the damaged state significantly reshaped the intestinal microbial community structure. The distance heatmap and UPGMA clustering tree further showed that all Lv-T samples clustered together and were distant from Lv-C samples, supporting a systematic difference in overall community structure between the high-alkaline group and the control group.

**Figure 10:**
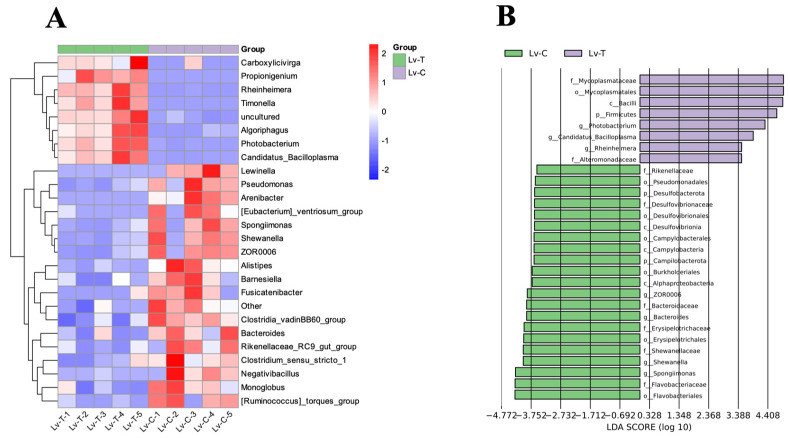
Differentially abundant genera and LEfSe analysis of the intestinal microbiota. (**A**) Heatmap displaying the relative abundance of all statistically significant differential genera (*p* < 0.05) between the treatment (Lv-T) and control (Lv-C) groups. (**B**) LEfSe analysis identifying the complete set of group-specific bacterial biomarkers (strictly filtered for LDA score > 2.0). High-resolution versions of these graphics are available in the online version of this article for detailed viewing.

As shown in Figure 10, at the genus level, comparison of the intestinal microbiota between the Lv-T and Lv-C groups identified 26 genera with significantly different relative abundances (*p* < 0.05). Among them, *Photobacterium*, *Candidatus Bacilloplasma*, *Rheinheimera*, and *Timonella* were markedly enriched in the Lv-T group: the mean relative abundance of *Photobacterium* was ~4.6% in the Lv-T group but only ~0.5% in the Lv-C group; *Candidatus Bacilloplasma* accounted for ~1.6% in the Lv-T group, but was almost undetectable in the Lv-C group; and *Rheinheimera* reached ~0.6% in the Lv-T group, while being close to 0 in the Lv-C group. Overall, these patterns indicate that, under alkaline conditions, opportunity-associated or highly host-dependent bacterial taxa, represented by Proteobacteria and some members of Firmicutes (e.g., the Firmicutes–Bacilli–Mycoplasmatales–Mycoplasmataceae-Candidatus *Bacilloplasma* and Proteobacteria–Gammapro-teobacteria–Alteromonadaceae–*Rheinheimera*/Vibrionaceae–*Photobacterium* lineages) increased significantly in relative abundance. By contrast, *Shewanella*, *Bacteroides*, *Spongiimonas*, as well as *Pseudomonas*, *Alistipes*, *Barnesiella*, ZOR0006, and *Monoglobus* were significantly enriched in the Lv-C group. The mean relative abundances of *Shewanella*, *Bacteroides*, and *Spongiimonas* were ~3.6%, 3.2%, and 6.6% in the Lv-C group, but only 1.3%, 1.6%, and 2.3% in the Lv-T group, respectively. These genera, together with their higher-level taxa, such as Bacteroidaceae, Rikenellaceae, Flavobacteriaceae, and Shewanellaceae, and the phyla Bacteroidota, Desulfobacterota, Campilobacterota, and Firmicutes-Erysipelotrichaceae, were identified by LEfSe as key biomarkers of the Lv-C group.

Taken together, the differential abundance and LEfSe results indicate that the damaged Lv-T group harbors an intestinal microbiota increasingly dominated by Proteobacteria/Firmicutes lineages, such as *Photobacterium*, Candidatus *Bacilloplasma*, and *Rheinheimera*, whereas the control Lv-C group maintains a community structure characterized by Bacteroidota-associated commensals, such as *Bacteroides*, *Spongiimonas*, and *Shewanella*, reflecting a typical shift from a ‘health-associated core microbiota’ toward a ‘damage-associated, opportunistic/host-dependent microbiota’ pattern.

## 4. Discussion

This study aimed to elucidate the effects of high-alkalinity stress on the antioxidant defense, immune performance, intestinal integrity, and gut microbial homeostasis of *L. vannamei*. To explore the underlying molecular mechanisms, we integrated physiological, histological, transcriptomic, and microbiome evidence. Through these complementary datasets, we sought to identify the key functional pathways mobilized during alkaline challenge. Ultimately, understanding these pathways reveals the organism’s inherent capacity and its limitations in maintaining homeostasis under environmental stress. Our results provide a mechanistic framework linking oxidative stress and energy allocation with immune suppression, tissue injury, and microbiota dysbiosis under high-alkalinity conditions. High-alkaline stress inhibited the antioxidant defense and immune function of *L. vannamei*. Under high-alkaline pressure, crustaceans produce numerous reactive oxygen species (ROS) as a result of respiratory inhibition [36]. During the initial stage, excessive ROS triggers the oxidative stress defense mechanism, leading to an increase in antioxidant enzymes [37,38]. However, the continuous accumulation of ROS results in oxidative damage [39], such as to mitochondria, as well as impaired aerobic respiration [40]. SOD is a component of the first line of defense against ROS and acts as a regulator of the immune function of *L. vannamei* [41,42]. An insufficient energy supply and the impact of ROS on various enzymes result in the decline of the activities of enzymes such as SOD, CAT, and GSH. Alkaline phosphatase (AKP) and acid phosphatase (ACP) are fundamental indicators for assessing immune function and health status, and are also important factors for acid–base regulation in crustaceans [43]. Both have significant roles in calcium absorption and phosphate calcium deposition [44], as well as in the destruction of pathogens [45]. Phenol oxidase (PO) is an important indicator for demonstrating the non-specific immune ability of *L. vannamei* [46]. The significant decrease in the activity of these enzymes in the treatment group indicates that high alkalinity caused significant damage to the immune system of *L. vannamei*. In addition, the respiratory burst activity and phagocytic index in the high alkalinity group significantly decreased, indicating that the immune phagocytic function is closely related to the energy supply capacity of the organism.

Transcriptome sequencing showed that, compared with the control group, the pathways related to exoskeleton synthesis and modification, transmembrane transport and signal initiation, and osmotic regulation were significantly activated in the high alkalinity group. Chitin is an important component of the exoskeletons of crustaceans and arthropods [47,48]. It is also involved in regulating various biological processes, such as molting and immune responses in crustaceans [49,50]. Chitinases are chitin-degrading enzymes with roles in growth, molting, development, and other biological processes [51]. Calcium signaling is a crucial factor in regulating the synthesis and release of molting hormones in crustaceans [52]. Under high-alkaline stress, the chitinase activity, chitin decomposition, and chitin-binding-related gene expression in *L. vannamei* were enhanced, indicating the formation of a new exoskeleton and facilitation of its growth and development under stress. Amino sugars are important substances for energy metabolism produced during glucose metabolism. They are involved in regulating intracellular signal transduction and cell recognition [53,54]. Nucleotide sugars are widely present in cells and are important metabolic products with a significant role in regulating signal transduction, cell division, and cell apoptosis [55,56]. Amino sugar and nucleotide sugar metabolism are metabolic pathways that promote energy production [57], thereby facilitating the adaptation of *L. vannamei* to high alkalinity.

High alkalinity causes osmotic imbalances in aquatic animals [2,18]. Amino acids can regulate osmosis through the oxidative energy supply and as organic osmotic substances [58]. In *L. vannamei*, the enhancement of β-alanine metabolism under high alkalinity could help it to regulate the osmotic balance in these two ways. Furthermore, the enhancement of transmembrane transporter activity also enables *L. vannamei* to enhance ion transport and promote osmotic balance. Therefore, alkaline stress can promote chitin metabolism and the expression of calcium-signaling-related genes in *L. vannamei*, providing a material basis for the synthesis of a new exoskeleton.

Compared with the control group, the *L. vannamei* in the high alkalinity group showed decreased expression of muscle contraction, a damaged muscular cytoskeleton, and ATP-binding-related functions. This suggests that *L. vannamei* has reduced energy metabolism and weakened movement ability under high-alkaline stress. Our results showed the reduced interaction between active neural ligands and receptors, which also impacts muscle inhibition, suggesting that energy is diverted from these functions to counter the high-alkalinity stress. Protein misfolding can lead to protein failure, which impacts the survival of cells. Protein refolding requires energy to repair incorrectly folded proteins [59]. Inositol phosphate is a storage depot for high-energy phosphate groups within cells and also serves as a messenger for the dynamic regulation of the cell skeleton [60]. Protein refolding and reduction in the inositol phosphate metabolic pathway indicate that *L. vannamei* has a decreased energy supply under high-alkaline stress, causing it to reduce physiological activities that are not essential for survival.

High-alkaline stress led to systematic damage of the midgut structure and barrier function in *L. vannamei*. The transcriptomic data revealed a significant downregulation in ATP-binding and muscular cytoskeleton pathways under high alkalinity, pointing to a severe energetic deficit. Because innate immune cascades (such as the proPO system) and mucosal barrier maintenance are highly energy-dependent, this forced reallocation of energy likely drives the downstream physiological phenotypes. Consequently, the reduced energetic capacity directly impairs the synthesis of acidic mucins and the integrity of the peritrophic membrane, while simultaneously constraining the effective activation of phenoloxidase (PO) and lysozyme (LZM) defenses against opportunistic pathogens. The high pH and ion concentrations in alkaline water disrupt normal physiological functions, leading to water loss and increased osmotic pressure [13], thereby causing damage to the tissues and even resulting in death. Under alkaline stress, the level of ROS in shrimp tissues increases [61], which can lead to tissue damage [62]. This structural dysbiosis, characterized by a shift toward Proteobacteria and Firmicutes lineages, is strongly associated with the compromised intestinal barrier observed in the high alkalinity group [63]. However, because these microbiome data are correlative, further germ-free or targeted microbial challenge models are required to establish whether this taxonomic shift is a direct cause or a secondary consequence of the environmental stress.

Shrimp intestines are rich in microbiota, and their function and stability are vital to the health of the host [64]. In this study, high-alkaline stress led to systemic changes in the gut microbiota of *L. vannamei*. High-alkaline stress significantly damages the intestinal mucosal epithelial cells of *L. vannamei*, thereby disrupting the structural integrity of the gut barrier. Consequently, the local expression of key antibacterial molecules is suppressed. This compromised mucosal defense facilitates the invasion of opportunistic and harmful bacteria, displacing beneficial commensal taxa and ultimately reducing overall microbial diversity [65]. The control group maintained a community structure characterized by Bacteroidota-related symbiotic bacteria. Bacteroidetes are involved in carbohydrate metabolism, nutrient absorption, immune regulation, and pathogen resistance [66]. The intestinal microbiota of the stress group became dominated by Proteobacteria and Firmicutes. Related studies have shown that Proteobacteria and Firmicutes can be used as probiotics to improve shrimp immunity by increasing the activities of proPO and superoxide dismutase [67]. This helps *L. vannamei* cope with the accumulation of peroxides caused by high-alkaline stress. However, studies have shown that an increased abundance of Proteobacteria is a potential disease risk [64]. Nevertheless, dysbiosis of the microbiota is a common sign that *L. vannamei* are in a stressed state. While our 16S rRNA sequencing reveals significant compositional shifts, these data strictly represent relative abundances. Without absolute quantification (e.g., via qPCR), it remains undetermined whether the observed expansion of opportunistic taxa, such as Photobacterium, represents absolute bacterial overgrowth or merely a proportional increase driven by the decline of previously dominant commensal populations. Future studies employing absolute quantification are required to resolve the exact population dynamics of the gut microbiota under alkaline stress.

## 5. Conclusions

This study systematically evaluated the effects of high-alkaline stress on the antioxidant defense, immune response, intestinal tissue structure, and gene expression regulation of *L. vannamei*. The results showed that high-alkaline stress significantly inhibited the antioxidant enzyme activity and non-specific immune function of *L. vannamei*, and caused damage to the intestinal mucosal barrier and disruption of the microbial community structure. At the same time, the stress activated the expression of genes related to chitin metabolism and ion transport, suggesting that *L. vannamei* responded to this stress through exoskeleton reconstruction and osmotic regulation mechanisms, accompanied by inhibition of energy metabolism and structural maintenance pathways. The results indicated that high-alkaline stress affected the physiological homeostasis of *L. vannamei* through oxidative stress and immune suppression, and the regulation of chitin synthesis and calcium signaling pathways could have a key role in the adaptation process. Unlike previous studies that have largely examined singular physiological stress markers, the originality of this work lies in its multi-omics integration. We provide novel evidence of a critical physiological trade-off: under high-alkalinity stress, the energetic demands forced by vital exoskeleton reconstruction and osmoregulation actively divert resources away from structural maintenance, directly causing the collapse of the intestinal mucosal barrier and the suppression of the innate immune cascade. Thus, this study provides a theoretical basis to explain the physiological response mechanism of *L. vannamei* in high-alkaline environments, and has guiding significance for the selection of salt-tolerant varieties and healthy breeding in saline-alkali water.

## Figures and Tables

**Table 1:** Growth indicators.

Group	Survival Rate (%)	Initial Body Length (cm)	Initial Weight (g)	Terminal Body Length (cm)	Terminal Weight (g)
2.5 mmol/L	93.33 ± 9.43 ^a^	12.58 ± 0.81	12.9 ± 1.16	12.79 ± 0.43 ^a^	13.62 ± 0.86 ^a^
5 mmol/L	80 ± 8.16 ^b^	12.58 ± 0.81	12.9 ± 1.16	12.69 ± 0.39 ^a^	13.27 ± 0.44 ^a^
10 mmol/L	63.33 ± 9.43 ^c^	12.58 ± 0.81	12.9 ± 1.16	12.64 ± 0.16 ^b^	13.02 ± 0.12 ^a^
20 mmol/L	53.33 ± 9.43 ^d^	12.58 ± 0.81	12.9 ± 1.16	12.61 ± 0.14 ^b^	13.07 ± 0.33 ^b^
40 mmol/L	43.33 ± 4.71 ^d^	12.58 ± 0.81	12.9 ± 1.16	12.54 ± 0.59 ^b^	12.66 ± 0.4 ^b^

Note: Different lowercase letters represent significant differences (*p* < 0.05).

**Table 2:** Primers used for RT-qPCR.

Primer	Sequences (5′–3′)	GenBank No.
ACP-F	ACATCTGTTCGTGGTTGC	KR676449.1
ACP-R	GGACTCGGATAATGCTCG	
AKP-F	GGCGGTCAGAGTGGAGAT	KR534873.1
AKP-R	CGCAATGCTGTAGAAGGAC	
LZM-F	TGCTGTTGTAAGCCACCC	AY170126.2
LZM-R	GTTCCGATCTGATGTCCG	
PO-F	AAGCCAGGCAGCAACCAC	XM_027381766.1
PO-R	CAGAAGTTGAAACCCGTGGC	
β-actin-F	TCCATGCCCAGGAATGAG	AF300705.2
β-actin-R	GAGCAGGAGATGACCACCG	

## Data Availability

The authors declare that all data supporting the conclusions of this study are available within the article.
